# Modelling the Costs and Effects of Selective and Universal Hospital Admission Screening for Methicillin-Resistant *Staphylococcus aureus*

**DOI:** 10.1371/journal.pone.0014783

**Published:** 2011-03-31

**Authors:** Gijs Hubben, Martin Bootsma, Michiel Luteijn, Diarmuid Glynn, David Bishai, Marc Bonten, Maarten Postma

**Affiliations:** 1 Department of Pharmacy, University of Groningen, Groningen, The Netherlands; 2 Department of Mathematics, Faculty of Science, Utrecht University, Utrecht, The Netherlands; 3 Department of Medical Microbiology, Julius Center for Health Sciences and Primary Care, University Medical Center Utrecht, Utrecht, The Netherlands; 4 BaseCase Software, Berlin, Germany; 5 School of Nursing, University of Ulster, Belfast, United Kingdom; 6 Johns Hopkins Bloomberg School of Public Health, Baltimore, Maryland, United States; Erasmus University Rotterdam, The Netherlands

## Abstract

**Background:**

Screening at hospital admission for carriage of methicillin-resistant *Staphylococcus aureus* (MRSA) has been proposed as a strategy to reduce nosocomial infections. The objective of this study was to determine the long-term costs and health benefits of selective and universal screening for MRSA at hospital admission, using both PCR-based and chromogenic media-based tests in various settings.

**Methodology/Principal Findings:**

A simulation model of MRSA transmission was used to determine costs and effects over 15 years from a US healthcare perspective. We compared admission screening together with isolation of identified carriers against a baseline policy without screening or isolation. Strategies included selective screening of high risk patients or universal admission screening, with PCR-based or chromogenic media-based tests, in medium (5%) or high nosocomial prevalence (15%) settings. The costs of screening and isolation per averted MRSA infection were lowest using selective chromogenic-based screening in high and medium prevalence settings, at $4,100 and $10,300, respectively. Replacing the chromogenic-based test with a PCR-based test costs $13,000 and $36,200 per additional infection averted, and subsequent extension to universal screening with PCR would cost $131,000 and $232,700 per additional infection averted, in high and medium prevalence settings respectively. Assuming $17,645 benefit per infection averted, the most cost-saving strategies in high and medium prevalence settings were selective screening with PCR and selective screening with chromogenic, respectively.

**Conclusions/Significance:**

Admission screening costs $4,100–$21,200 per infection averted, depending on strategy and setting. Including financial benefits from averted infections, screening could well be cost saving.

## Introduction

*Staphylococcus aureus* is one of the most common causes of nosocomial and community-acquired infections. Since the 1980s, methicillin-resistant *S. aureus* (MRSA) nosocomial prevalence levels have increased in most countries [1]–[3]. An estimated 25,100 nosocomial MRSA infections occurred in the US in 2005 [4], and have been associated with higher costs, higher mortality and an increased length of stay than infections with methicillin-susceptible *S. aureus* (MSSA) [5], [6].

The low nosocomial prevalence in Scandinavian countries and the Netherlands has been ascribed to stringent policies to control the spread of MRSA. Bootsma et al. have investigated the contribution of different components of the Dutch *Search and Destroy* policy [7], indicating that admission screening can effectively reduce MRSA in high prevalence settings [8]. In clinical studies selective screening on admission to intensive care units (ICUs) or universal screening at hospital admission yielded conflicting results [9]–[15]. Universal admission screening might be an economically viable option through prevention of MRSA infections and its associated costs [15], but has not been widely adopted because of the presumed high costs associated with testing and subsequent isolation [16].

Several detection tests are now commercially available, each with different test characteristics and costs. The impact and relative importance of a test's sensitivity, specificity and test delay depend on the screening strategy used and the MRSA prevalence in the catchment population. Here, we used a modeling approach to assist hospital administrators in informed decision making on the implementation of an admission screening strategy.

The objectives were (1) to estimate the costs of screening and isolation per infection averted for various admission screening strategies, (2) to compare two MRSA detection tests within these strategies and (3) to investigate the relative importance of test sensitivity, specificity and test delay. Our analysis focused on the United States.

## Study design

We performed an analysis of costs and effects of universal and selective MRSA screening at hospital admission, combined with isolation of identified MRSA carriers, over a timeframe of 15 years, using a 3% annual discount rate [17]. We compared strategies both to each other and to a baseline without screening or isolation. The analysis was conducted from a US hospital's perspective, and costs are reported in US dollars using price levels of the year 2007.

We used a previously published [8] discrete event simulation model developed with C++, reflecting MRSA transmission within hospitals, to estimate the health and economic outcome of screening and isolation. The incremental cost-effectiveness ratio (iCER) of selected strategies was calculated as the difference in screening and isolation costs divided by the difference in infections, of one strategy over another. We also present the average cost effectiveness ratios (aCERs) for each strategy, calculated as the costs of screening and isolation costs divided by the difference in MRSA infections, relative to a baseline of no screening and no isolation. As our main outcome measure is the investment costs per infection averted, we counted up-front investment costs of screening and isolation (e.g., lab tests and contact precautions), but excluded cost consequences of averting MRSA infection, such as a shorter hospital stay and averted treatment costs. Instead, we compare estimated investment costs with financial benefits of averted MRSA infections.

## Overview of the simulation model

Below, we present a brief overview of the model, a more detailed account is available elsewhere [8]. Parameter estimates are based on data obtained in the University Medical Center Utrecht, the Netherlands, unless specified otherwise. The model simulates three hospitals, each with 693 beds (36 18-bed wards and 5 9-bed intensive care units (ICUs)) with a 100% bed-occupancy. The mean length of stay was assumed at 3 and 7 days within ICUs and regular wards, respectively (exponentially distributed). Each hospital has a catchment population of 220,000 individuals, of which 20,000 are known ‘high risk’ individuals that have a 10 times higher probability of being admitted to the hospital, compared to the non-‘high risk’ individuals. This leads on average to a hospital population of 50% ‘high risk’ and 50% non-‘high risk’ patients. Additionally, ‘high-risk’ patients are characterized by a life expectancy of 20 years versus 78 years for non-‘high-risk’ patients. One can think of the high-risk group as elderly together with immunocompromised patients. Unidentified hospitalized carriers have a daily probability of 3% of being detected through conventional microbiological cultures obtained for clinical reasons [8]. Individuals identified as MRSA carrier during a hospitalization are ‘flagged’, so that they are identified as such on a next admission.

MRSA transmission occurs primarily via patient-to-patient transmission mediated by the hands of health care workers (HCWs). The adherence of HCWs to the hand-washing protocol is assumed to be constant over time. Transmission is 20 times more likely to occur within a given hospital unit, compared to transmission between units. Transmission can also occur via HCWs who are colonized in the nose/throat [18]. In a high prevalence setting, this route is set to be 8 times less important as patient-to-patient transmission. Finally, the transmission rate in ICUs is assumed to be 3 times higher (for both routes) compared to other wards, due to more frequent contacts between HCWs and patients and the higher susceptibility of ICU patients. The transmission parameters were calibrated to obtain a steady-state nosocomial prevalence of 15% at baseline (high prevalence).

We used an average daily probability of developing an infection of 0.59% for a hospitalized carrier [19]. Coello et al. report that half of the 68 infections occured within 12 days. We can derive a daily probability of 0.59% by dividing the number of infection (68/2 = 34) by the total time at risk (479 patients * 12 days  = 5748 days). This results in an infection rate of 8.9 per 10,000 bed days at baseline with 15% nosocomial prevalence. Infection status was not explicitly modeled and, therefore, infected patients had the same infectiousness and discharge probabilities as MRSA carriers. We evaluated all screening strategies in a high and medium nosocomial prevalence setting of initially 15% [20]–[23] and 5% [24], respectively. This prevalence is defined as the percentage of positive findings when performing a cross sectional screening of all patients in the hospital with a perfect test. For the high prevalence setting, the screening program was initiated after a simulation time of 10 years. This period was used to avoid major effects of the exact initial conditions and to reach a steady state nosocomial prevalence of 15%. This prevalence level corresponds to 5.5% prevalence upon hospital admission. In the medium prevalence setting, the simulations were started using a prevalence <1%, and the screening program was initiated when the average nosocomial prevalence in the three hospitals reached 5% for the first time. The outcome of our stochastic model is presented for one hospital with 693 beds, as the mean of 1000 simulations for each strategy over the full timeframe of 15 years. The 2-sided 95% uncertainty intervals (UIs) cover the results observed in 95% of the simulations.

## Baseline

At baseline there is neither active screening for MRSA nor isolation of identified or suspected carriers. The nosocomial prevalence remained at a steady state of 15% over the entire time frame in high prevalence settings. As a baseline for the medium prevalence setting, we assumed a steady-state prevalence of 5% over the time frame, although without interventions the prevalence would continue to rise to the high prevalence level.

## Admission screening and isolation

We evaluated ‘Selective’ screening of ‘high risk’ patients and ‘flagged’ patients only, as well as ‘Universal’ screening of all patients. Both strategies were evaluated with a PCR-based test and a chromogenic media-based test (see table 1 for test characteristics). We define test delay as the time between collection of specimens and the reporting of results to the wards, which includes transport and laboratory time. We assumed a test delay of 0.5 day for PCR, and 1.5 and 2.5 days for the chromogenic media-based test after 24 and 48 hours of incubation, respectively. One swab is taken from patients at admission which is subsequently tested for MRSA, without confirmation by conventional culture techniques. Identified MRSA carriers are isolated in single rooms, and are not decolonized during their hospital stay. We assumed no limits on isolation capacity to allow the peak isolation capacity required for each screening strategy to be determined by the model.

**Table 1:** Test characteristics.

Test	Sensitivity [28]	Specificity [28]	Test delay (days)
PCR	92.5	97.0	0.5
Chromogenic^1^			
At 24 h	78.3	98.6	1.5
At 48 h	87.6	94.7	2.5

1 The chromogenic media-based test is evaluated after 24 and 48 hours of incubation. Patients with positive results are isolated at both time points, with the last result after 48 hours being considered final.

## Base-case assumptions

To simulate a regionally implemented MRSA screening policy, all three hospitals in the model are assumed to implement identical screening strategies at the same time. The chromogenic media-based test is evaluated after 24 and 48 hours of incubation. Patients with positive results are isolated at both time points, with the last result after 48 hours being considered final. Pre-emptive isolation, defined as isolation upon readmission for the duration of the test delay until confirmed negative for carriage of MRSA, is limited to ‘flagged’ patients only. Single room isolation is assumed to reduce the risk of transmission by 80% [8].

## Scenario analysis

We additionally investigate four alternatives to our base-case assumptions: (1) full pre-emptive isolation, that includes pre-emptive isolation for ‘high risk’ as well as ‘flagged’ patients; (2) the absence of pre-emptive isolation; (3) only 1 out of the 3 hospitals in the model implements screening; (4) screening with a chromogenic media-based test, using only the results after 24 h of incubation.

## Cost data

The total investment cost borne by the hospital is assumed to consist of the additional cost of isolation plus the cost of screening. The screening and isolation costs were calculated by multiplying estimated resource use (including labor) by unit prices (table 2) (source: bureau of labor statistics, US department of labor). The prices of consumables were provided by the manufacturers. The costs of isolation were calculated assuming that facilities for single room isolation are available, thereby excluding the capital costs of building new infrastructure. The isolation costs consist of contact precautions and additional cleaning of the room in case of a positive screening test. The costs of the screening program consist of tests, laboratory labor, laboratory equipment, labor of taking swabs and of a clinical risk assessment when screening selectively (table 2).

**Table 2:** Resource use and costs of screening and isolation in US$ (2007).

Item	Units	Costs ($)
*Screening*		
Take swab by nurse [13]	5 (min)	3.1
Clinical risk assessment by nurse^1^	5 (min)	3.1
Transport swab	1	0.35
**Fixed screening costs**		**6.55**
*Screening – PCR*		
PCR - test cost per sample	1	24.0
PCR - test clinical lab. technician time per sample [34]	1.5 (min)	0.76
Fixed screening costs		6.55
**Total cost per patient**		**31.3**
PCR - annual cost real-time PCR equipment 2	1	4,315
*Screening – Chromogenic*		
Chromogenic - test cost per sample	1	3.5
Chromogenic - clinical lab. technician time per sample [35]	11.1 (min)	5.6
Fixed screening costs		6.55
**Total cost per patient**		**15.7**
*Isolation*		
Contact precautions materials per day^3^	12	12.4
Contact precautions additional nurse time per day [*11*]	36 (min)	22.3
Contact precautions additional physician time per day [*9*]	10 (min)	13.7
**Total cost per patient**		**48.4**
Cleaning of room 4	30 (min)	7.4

1 The time required to estimate the risk of being a carrier was based on factors such as hospital admission within last 12 months or transfer from another healthcare facility (only in case of selective screening).

2 Annual cost based on Smartcycler (Cepheid, Sunnyvale, CA), straight line depreciation using an interest rate of 4%, a cost of $35,000, a lifetime of 10 years and a resale value of 20%.

3 Total $1.04, including gloves ($0.057), gown ($0.46), mask ($0.27), hair cap ($0.049), disinfectant 75 mL ($0.20) required for each of 12 entries into an isolation room per day.

4 Additional cleaning costs are only incurred in case of a positive finding.

Labor costs are based on nationwide average hourly wages for registered nurses ($29.8), physicians ($66.3), clinical laboratory technologists and technicians ($24.4) and janitors and cleaners ($11.9). (source: bureau of labor statistics, US department of labor). A 24.3% administration overhead was applied to all labor costs [*36*]. Prices of consumables were provided by manufacturers.

## Sensitivity analysis

In a one-way sensitivity analysis we investigated the impact of alternately varying the test sensitivity (50–100%), specificity (50–100%) and test delay (0–5 days), on the costs and infections averted. Additionally, we investigated the impact of varying key model parameters on the aCER. The sensitivity analysis was conducted using the strategy selective screening with PCR in a high prevalence setting.

## Results

### Screening strategies

**Figure 1:**
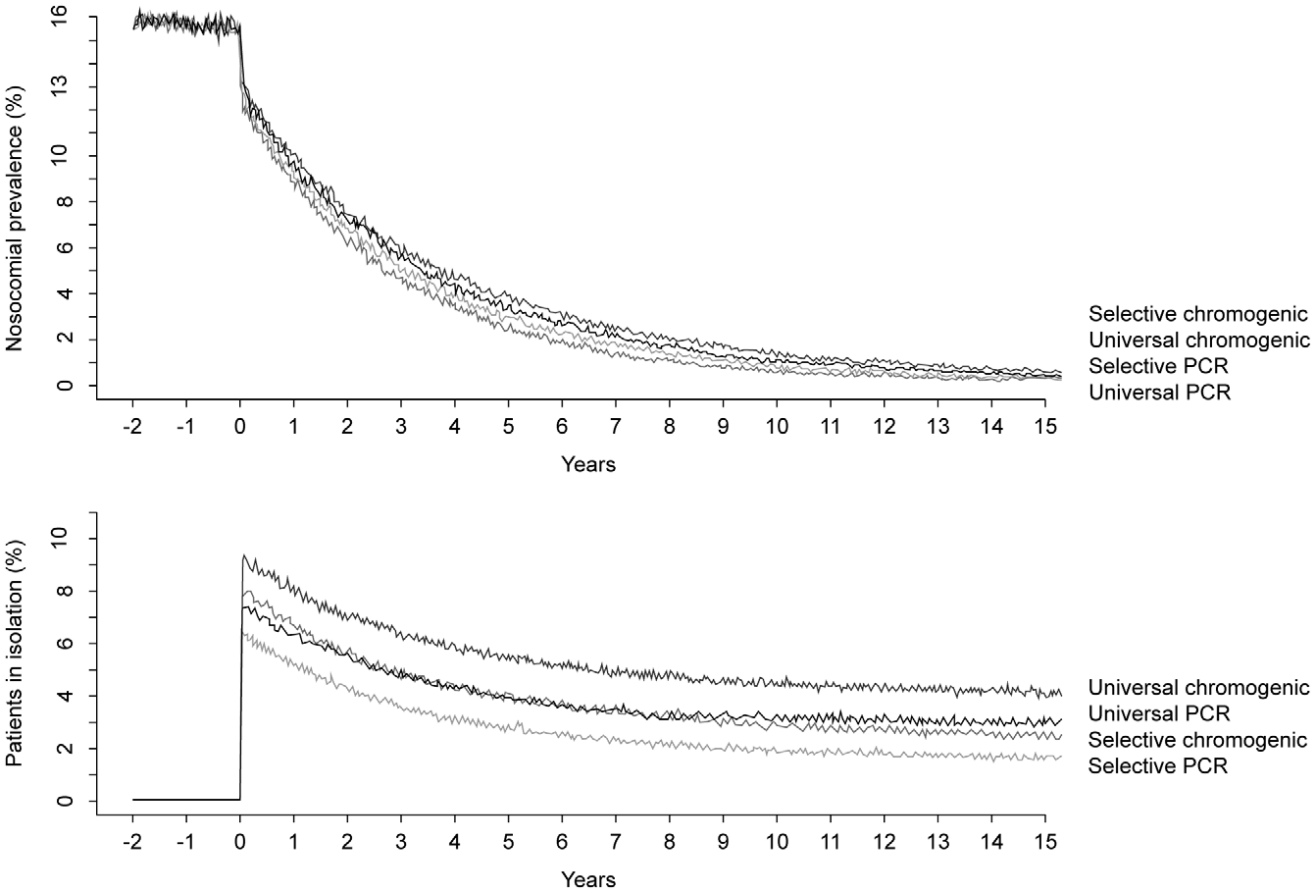
Nosocomial prevalence and patients in isolation over time. The upper graph shows the impact of the screening strategies on the nosocomial prevalence over time. The lower graph shows the percentage of total patients in isolation over time for each strategy. Both graphs show the mean of 1000 runs of the model.

Relative to baseline, all strategies reduced MRSA prevalence in the first years of screening, yielding prevalence rates below 1% after 15 years (figure 1). The number of patients screened over this period was roughly 200,000 and 400,000 per hospital for selective screening and universal screening, respectively.

**Figure 2:**
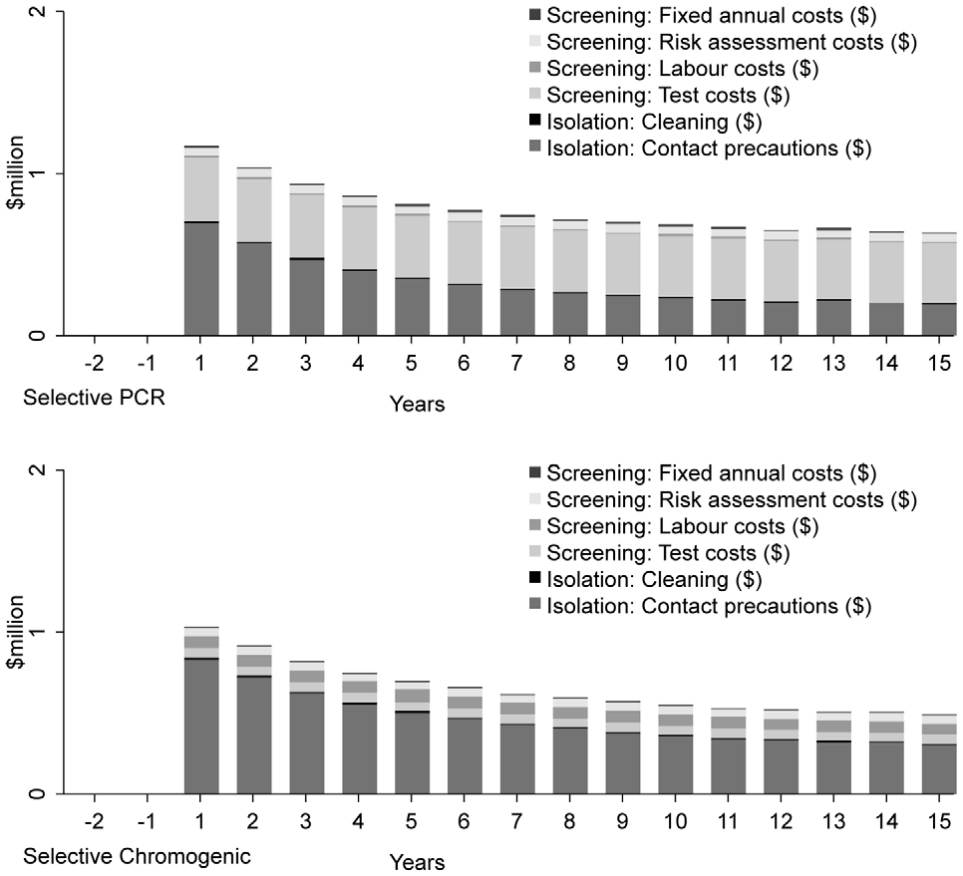
Annual cost of screening and isolation, and rate of infection. The annual undiscounted cost in US$ (2007) of strategies ‘Selective PCR’ (left) and ‘Selective Chromogenic’ (right) in a high prevalence setting. The first two years represent baseline (no screening and no isolation).

Percentages of patients in isolation over time are characterized by a peak at the start of the screening program (figure 1). The peak percentage of patients in isolation ranged from 6.2% to 9.1% and 2.9% to 5.0% for high and medium prevalence, respectively, and was higher for universal screening than for selective screening (table 3). The annual costs associated with screening and isolation decrease over time, and are shown for ‘Selective PCR’ and ‘Selective Chromogenic’ in a high prevalence setting (figure 2). The screening costs of PCR testing are higher than for chromogenic testing, but these costs are partially offset by the lower costs of isolation of ‘Selective PCR’.

**Table 3:** Results of screening strategies.

Strategy	Test	Screening ($m)	Isolation($m)	Total Investment Cost ($m)	Cases of infection	Cases of infection averted vs. baseline	aCER (Total investment cost $ per infection averted) (95% UI)	Isolation^1^	Peak isolation capacity required (%)^2^	Patients screened	Time to 50% prevalence reduction (Yrs)^3^	Prevalence after 15 years (%)
**High**												
Baseline	None	0	0	0	2753	0	NA	0	0	0	NA	15
Selective	PCR	6.17	4.05	10.22	547	2,206	4,633 (4,477–4,843)	83,774	6.2	200,179	3.46	0.28
Selective	Chromogenic	2.87	5.78	8.65	668	2,085	4,149 (3,948–4,442)	119,407	7.2	200,839	3.92	0.49
Universal	PCR	10.42	5.89	16.30	501	2,252	7,237 (7,000–7,487)	121,681	7.8	375,725	3.33	0.22
Universal	Chromogenic	4.21	8.15	12.36	622	2,131	5,799 (5,484–6,142)	168,449	9.1	375,739	3.73	0.42
**Medium**												
Baseline	None	0	0	0	918	0	NA	0	0	0	NA	5
Selective	PCR	5.81	2.71	8.52	237	681	12,508 (11,454–13,677)	55,981	2.9	188,374	4.19	0.20
Selective	Chromogenic	2.69	3.69	6.38	296	622	10,257 (9,110–11,819)	76,226	3.3	188,461	4.96	0.33
Universal	PCR	10.42	4.61	15.03	209	709	21,195 (19,841–23,347)	95,310	4.3	375,745	3.87	0.17
Universal	Chromogenic	4.21	6.18	10.39	271	647	16,056 (14,593–18,106)	127,664	5.0	375,766	4.58	0.27

1 The number of patient days in isolation.

2 The peak isolation capacity required by the hospital in 97.5% of all simulations.

3 The number of years required to reach a 50% reduction in the nosocomial prevalence.

The cumulative and discounted costs in US$ (2007) and discounted effects for one hospital over 15 years, using base-case assumptions, for a high (15%) as well as a medium (5%) prevalence setting.

**NA** not applicable; **aCER** average cost-effectiveness ratio in $ per infection averted, compared to no screening; **UI** uncertainty interval;

The total number of infections at baseline - over the 15 year timeframe - amounted to 2,753 and 918 for high and medium prevalence, respectively. Of these infections, the number averted by the different screening and isolation strategies ranged from 2,085 to 2,252 and from 622 to 709 for high and medium prevalence, respectively (table 3).

The least costly strategy in terms of the costs per infection averted is ‘Selective Chromogenic’. The investment costs of this strategy in a high prevalence setting are $8.7 m and it averts a total of 2,085 (2,085/2,753 = 76%) infections compared to baseline (table 3). In a medium prevalence setting, ‘Selective Chromogenic’ costs $6.4 m and averts 622 (622/918 = 68%) infections compared to baseline.

The most effective strategy was ‘Universal PCR’, averting 2,252 (82%) and 709 (77%) infections in high and medium prevalence settings, respectively. This strategy was also the most costly, requiring a total investment of $16.3 m and $15.0 m for high and medium prevalence, respectively.

**Figure 3:**
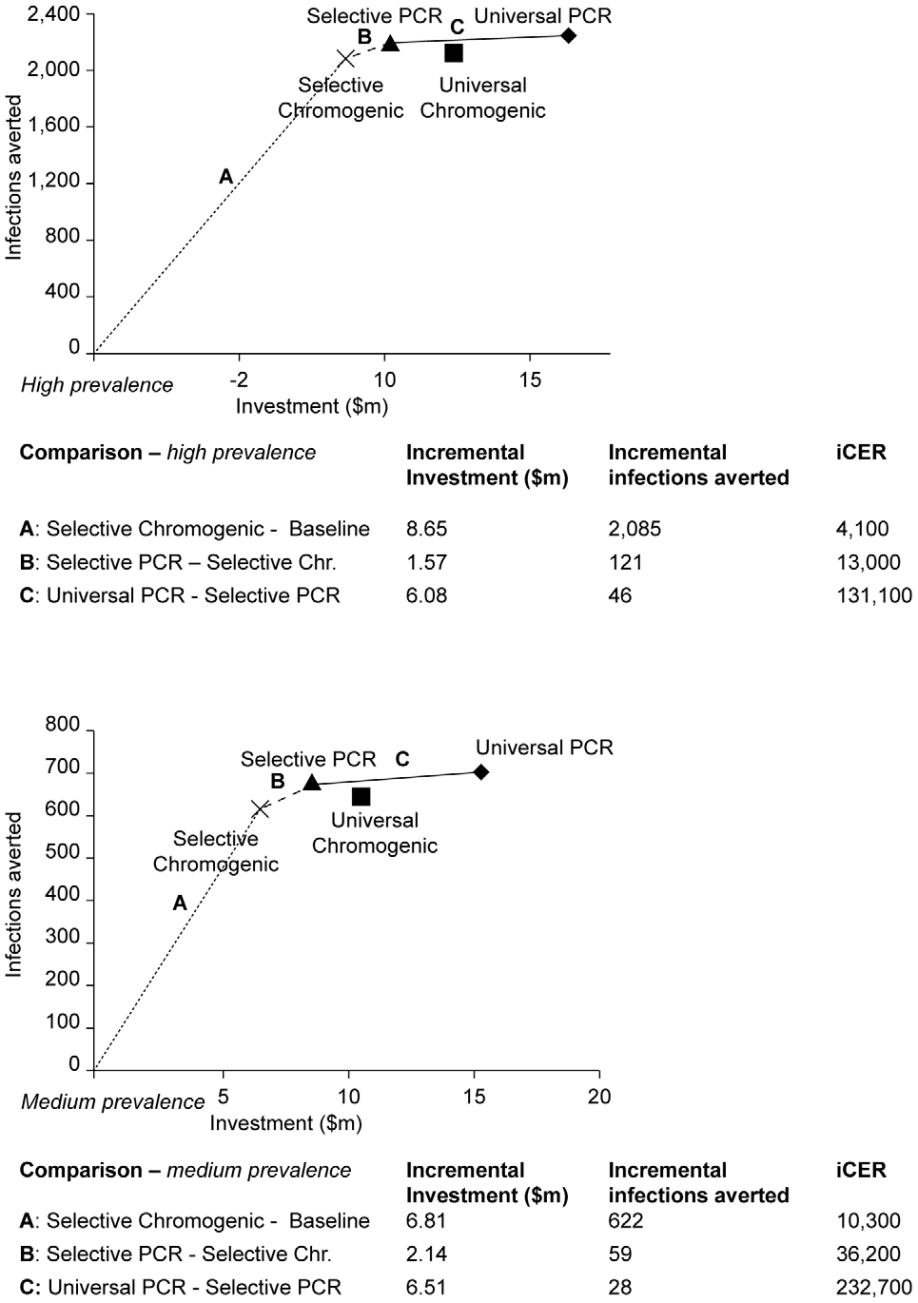
Cost effectiveness planes for the high (top) and medium (bottom) prevalence setting. The investment costs in millions in US$ (2007) are depicted on the horizontal axis and health benefits (infections averted) on the vertical axis. The points shown represent the infections averted and investment costs of each screening strategy. The origin represents baseline, a policy of neither screening nor isolation. The incremental ratios of D effectiveness to costs are represented by the slopes of the lines connecting these points. The decreasing slope illustrates the diminishing return on investment when extending the selective PCR to universal screening in both settings. The strategy ‘Universal Chromogenic’ is dominated by ‘Selective PCR’ (higher costs, less health benefits), and is therefore not considered a relevant option. The incremental investment costs, infections averted and incremental cost-effectiveness ratio between selected strategies are shown in the table beneath the graphs. **iCER** incremental cost-effectiveness ratio; **Chr**. Chromogenic.

To visualize comparisons between strategies, we plotted costs and health gains of each strategy (figure 3). In the high prevalence setting, the aCER of selective screening of ‘high risk’ patients with a chromogenic media-based test (‘Selective Chromogenic’), compared to baseline, is $4,100 per infection averted, which is represented by line A. Substituting the chromogenic media-based test by a PCR-based test (‘Selective PCR’), represented by line B, costs an additional $1.6 m and averts 121 more infections, resulting in an iCER of ‘Selective PCR’ compared to ‘Selective Chromogenic’ of $13,000 per additional infection averted. An extension of ‘Selective PCR’ to all patients (‘Universal PCR’), costs an additional $6.1 m and averts an additional 46 infections, resulting in an iCER of $131,000 per infection averted (line C).

In the medium prevalence setting, the aCER - compared to baseline – of screening ‘high risk’ patients with a chromogenic based test (‘Selective Chromogenic’) is $10,300 per infection averted. Substituting the chromogenic media-based test by a PCR-based test (‘Selective PCR’), represented by line B, costs an incremental $2.1 m and averts an incremental 59 infections, resulting in an iCER of ‘Selective PCR’ compared to ‘Selective Chromogenic’, of $36,200 per additional infection averted. The incremental returns on investment strongly diminish with an extension of ‘Selective PCR’ to all patients (‘Universal PCR’), at an iCER of $232,700 per additional infection averted (line C).

Universal screening with a chromogenic media-based test is dominated in both settings by selective screening with PCR (i.e. selective screening with PCR is both cheaper and more effective).

### Scenario analysis

Comparing selective screening with PCR using base-case assumptions with the individual scenarios (table 4), shows that extending pre-emptive isolation from ‘flagged’ patients only to all ‘high risk’ patients, averts 35 (+1.4%) additional infections at an additional cost of $3.6 m (+34.9%). The absence of any pre-emptive isolation reduces the number of infections averted by 32 (−1.4%) and costs by $0.4 m (−4.3%). If only one out of the three hospitals implements screening, the total investment costs are $ 0.8 m (7.8%) higher than the total investment costs of the 3 hospitals in base case scenario, while the number of infections averted in the participating hospital diminishes by 254 (−11.5%) (158 infections are averted in each of the non-participating hospitals). A screening program using only the results of the chromogenic media-based test at 24 h of incubation, reduces the number of infections averted by 211 (−9.6%) and also costs by $3.6 m (−35.7%), compared to PCR-based screening.

**Table 4:** Results of the scenario analysis.

Strategy	Test	Screening ($m)	Isolation($m)	Total Investment Cost ($m)	Cases of infection	Cases of infection averted vs. baseline	aCER (Total investment cost $ per infection averted) (95% UI)	Isolation^1^	Peak isolation capacity required (%)^2^	Patients screened	Time to 50% prevalence reduction (Yrs)^3^	Prevalence after 15 years (%)
Baseline	None	0	0	0	2753	0	NA	0	0	0	NA	15
**Base-case scenario** Selective –preemptive isolation of ‘flagged’ patients	PCR	6.17	4.05	10.22	547	2,206	4,633 (4,477–4,843)	83,774	6.2	200,179	3.46	0.28
**Scenario 1** Selective –Full preemptive isolation	PCR	6.17	7.63	13.80	512	2,241	6,158 (5,920–6,406)	157,568	8.3	200,176	3.37	0.22
**Scenario 2** Selective –No preemptive isolation	PCR	6.17	3.62	9.79	579	2,174	4,502 (4,298–4,703)	74,714	5.5	200,178	3.58	0.37
**Scenario 3** Selective –1 out of 3 hospitals screens	PCR	6.24	4.79	11.02	801	1,952	5,646 (5,232–6,086)	98,900	5.8	202,360	3.50	2.59
**Scenario 4** Selective – Chromogenic media-based test after 24 h of incubation	Chromo-genic 24	2.87	3.70	6.58	758	1,995	3,299 (3,076–3,555)	76,543	5.9	200,730	4.23	0.80

1 The number of patient days in isolation.

2 The peak percentage of total patients in isolation in 97.5% of all simulations.

3 The number of years required to reach a 50% reduction in the nosocomial prevalence.

The cumulative and discounted costs in US$ (2007) and discounted effects for one hospital over 15 years, for a high (15%) prevalence setting.

**NA** not applicable; **aCER** average cost-effectiveness ratio in $ per infection averted, compared to no screening; **UI** uncertainty interval;

### Sensitivity analysis

**Figure 4:**
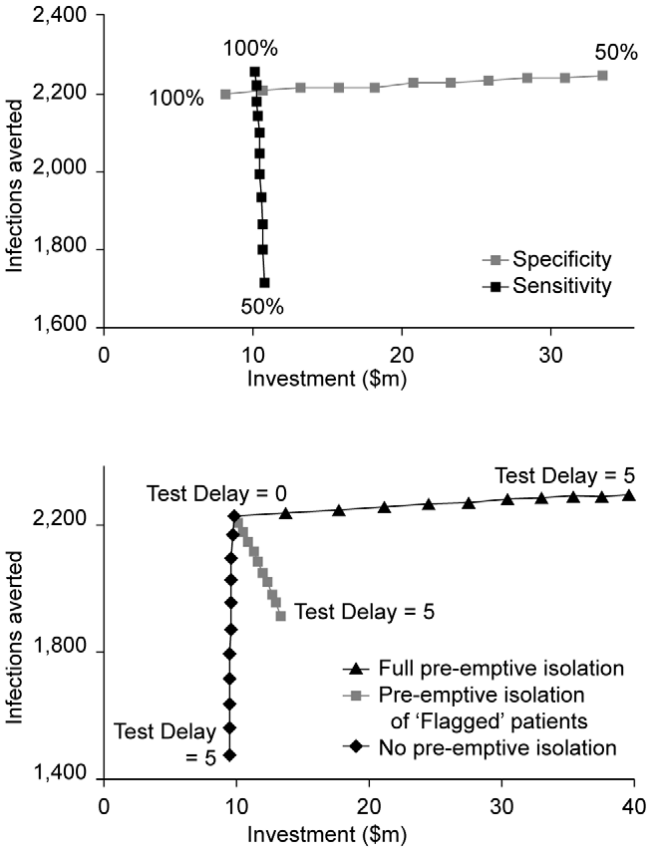
Results of the sensitivity analysis of test characteristics. The costs of selective PCR-based screening are depicted on the horizontal axis and health benefits (infections averted) on the vertical axis. The left graph shows the combined results of alternately varying the test’s sensitivity and specificity from 50% to 100%, with increments of 5%. The right graph shows the test delay varied from 0 to 5 days, with increments of 0.5 day, for different pre-emptive isolation strategies: No pre-emptive isolation (diamonds), pre-emptive isolation of ‘flagged’ patients only, i.e. the base-case scenario (squares), and full pre-emptive isolation, i.e. ‘flagged’ patients as well as ‘high risk’ patients (triangles).

**Figure 5:**
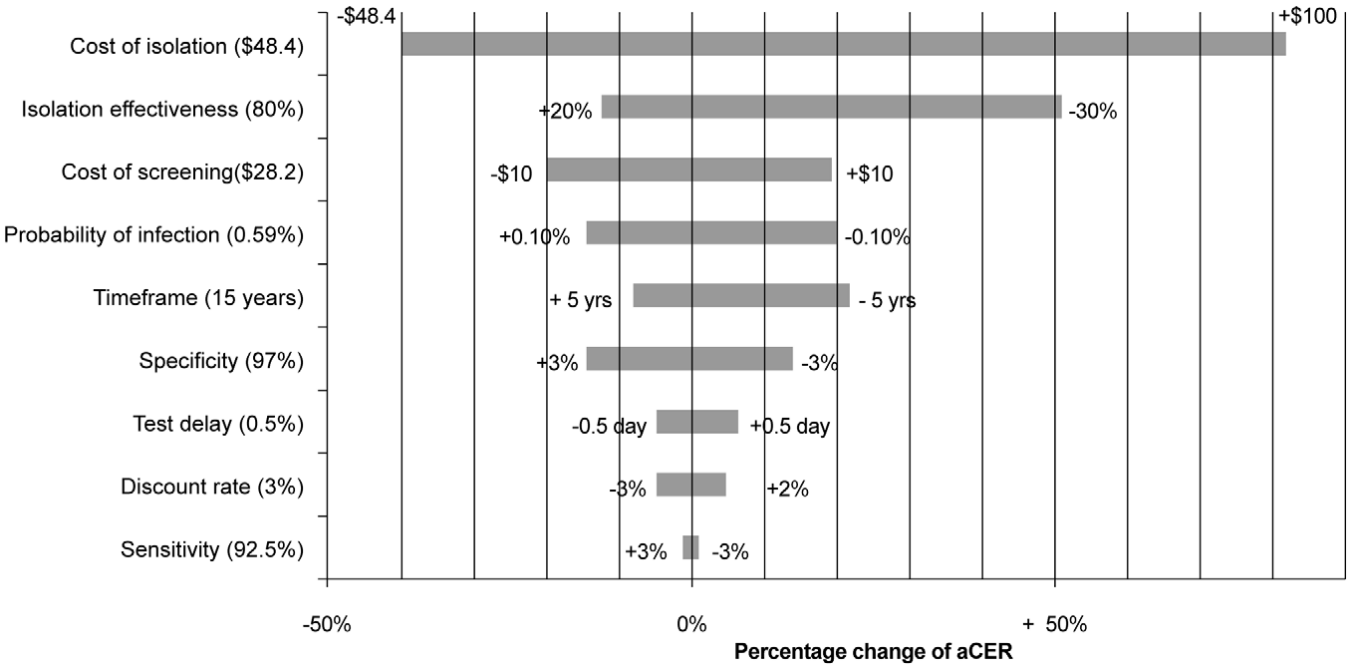
Results of one-way sensitivity analysis on key model parameters. Parameters are ranked by the magnitude of their impact on the average cost-effectiveness ratio (aCER), of selective screening with PCR (aCER: $4,600) under base-case assumptions (base-case parameter values are shown between brackets).

The investment costs and the infections averted of varying test sensitivity and specificity from 50% to 100% with increments of 5%, are shown in figure 4 (left panel). A higher test sensitivity increases the number of infections averted but has very little impact on costs. A higher specificity strongly reduces costs but has a minor impact on health outcome. The slight increase in infections averted with a decreasing specificity is caused by the higher number of patients that are isolated based on a false positive test result and are therefore at lower risk of transmission. The right panel of figure 4 shows the impact of varying the test delay from 0 to 5 days. For our base-case scenario a higher test delay reduces the number of infections averted and also increases costs. Different levels of pre-emptive isolation change the impact of the test delay. When using ‘full preemptive isolation’, an increasing test delay causes a slight increase in the number of averted infections. This can be attributed to the effect of isolating all high risk patients (∼50% of all hospital admissions) for a substantial part of their hospital stay. Figure 5 shows key model parameters ranked by the magnitude of their impact on the aCER. Additionally we investigated the impact of commonly used discount rates for costs and effects: relative to baseline, a discount rate of 4% for both costs and effects resulted in aCER increase of 2%. A combination of a discount rate of 4% for costs and 1.5% for effects resulted in a reduction in the aCER of 16%.

## Discussion

### Cost savings

**Figure 6:**
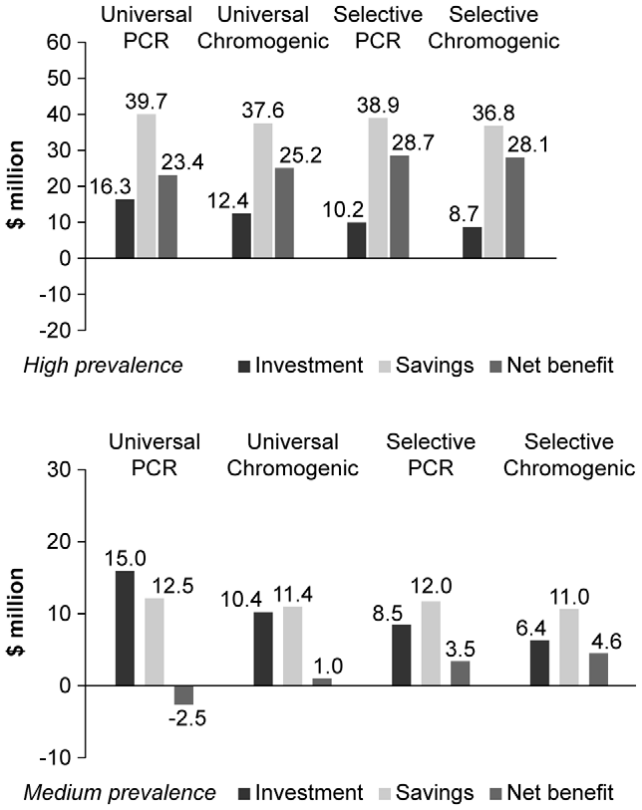
Total investment costs, savings and net benefit of strategies. Investment costs, savings (based on $17,645 averted hospital costs per averted infection ([6]) and net benefit in millions of US$ (2007), in high (left) and medium (right) prevalence settings.

The true costs attributable to MRSA infection are unknown and the appropriate method to determine these costs is debated [16]. Reported additional hospital costs of MRSA infection over no infection range from $6,709 to $64,216, depending on the type of infection [6], [9], [15], [25]. Additional hospital costs of MRSA infections over MSSA infections range from $8,327 to $16,738, depending on the type of infection [6], [25], [26]. Using a recently published estimate of hospital costs ($17,645 translated to US$ 2007) of MRSA infection over no infection [6], we can compare the financial benefits of averted infections to the investment costs per infection averted, and estimate the net benefits (figure 6). If the averted hospital costs of infection are real savings to the hospital, all evaluated screening strategies are cost-saving in a high prevalence setting. The net benefit is estimated at $28.7 m for ‘Selective PCR’, and $28.1 m for ‘Selective Chromogenic’, followed by $25.2 m for ‘Universal Chromogenic’ and $23.4 m for ‘Universal PCR’. In a medium prevalence setting, the net benefits are lower; $4.6 m for ‘Selective Chromogenic’ and $3.5 m for ‘Selective PCR’, followed by $1.0 m for ‘Universal Chromogenic’. ‘Universal PCR’ was not cost-saving in this setting with a net benefit of $−2.5 m.

### Additional considerations

Our scenario analysis confirms that admission screening will be less effective and more costly if neighboring hospitals do not screen [27]. The lack of effective regulation to ensure regional compliance with MRSA screening inhibits single hospitals from screening because it is less cost-effective to be the only screening hospital in the region. It also demonstrates that extensive pre-emptive isolation is a relatively costly infection control strategy when a test with a low test delay is used. By using a chromogenic media-based test after 24 h of incubation, the isolation costs can be reduced, because of the shorter test delay and the higher specificity, but results in fewer infections averted.

When extending the time frame, the costs per infection averted decrease (figure 5), because the declining prevalence of each additional year is compared to the higher baseline prevalence. A lower isolation effectiveness (50% instead of 80%), which might be due to less effective use of barrier precautions or hand hygiene, or reflect the potential of transmission while in cohort isolation if precautions are not followed well, increases the aCER by 50%. The model outcome is very sensitive to the cost of isolation. If we assume that the additional costs of single room isolation are on average $100 per day, the aCER increases more than 80%. Higher additional isolation costs benefit strategies with a short test delay. At thresholds for isolation costs of $25 and $51 for high and medium prevalence settings respectively, ‘Selective PCR’ becomes more cost-effective than ‘Selective Chromogenic’, and at thresholds of $45 and $106 for high and medium prevalence settings respectively, ‘Selective PCR’ becomes dominant over ‘Selective Chromogenic’ (i.e. less costly and more effective).

Our results contrast with another recent economic analysis [13], which recommended screening with a chromogenic media-based test after 24 hours of incubation over PCR-based screening. An important difference is that this study assumed an equal test delay of 1 day for both tests, and used a sensitivity and specificity for the chromogenic media-based test of 98.0% and 99.8%, respectively, where we have used 76.6% and 98.6%, based upon a recently performed meta-analysis [28].

### Study limitations

The outcomes from our study depend on the validity of the transmission model. To assess the validity of our model we conducted extensive sensitivity analyses and have provided estimates around our estimated aCER. We did not perform a full probabilistic sensitivity analysis to estimate the impact of the uncertainty in the assumed model parameter values, because the computation time required would be unfeasibly long for the type of model we used. Instead, the impact of varying model parameters was investigated using one-way sensitivity analysis.

Because a model remains a simplification of real life situations, the inherent limitations should be discussed. No limit was set on isolation capacity and it was assumed that all identified carriers were isolated, with corresponding isolation costs. However, this ideal policy will not always be realized [29]. Failure to isolate will reduce the total isolation effectiveness, but will also reduce costs. In our analyses we considered isolation not to be perfect (80% reduction in infectiousness), but costs were always incurred. This will overestimate the costs per infection averted. We assumed an average rate of infection for all carriers, whereas this rate may differ between patients in ICU and in a regular ward.

There are no published estimates on the additional cost (if any) of a patient in a single room versus a semi-private room or a ward [30], and consequently we have omitted these costs from our analysis, as others have done [9], [15]. Some authors have included estimates based on construction costs [9], on the maintenance of the additional floor space required [13], or on revenue lost [31]. These approaches can be valid but are strongly determined by local conditions, such as the type of infrastructure, the shared use of isolation facilities for other pathogens and the level of hospital occupancy. Some additional opportunity costs are likely to occur in a hospital operating at near full capacity, due to bed blocking [32]. We have used sensitivity analysis to estimate the impact of additional single room isolation costs.

As our main outcome measure was investment costs per infection averted, our calculations neglect the benefit of patients of not having MRSA. The healthcare utilization costs of treating MRSA infection are driven by the patient’s length of stay. The length of stay varies considerably across hospitals and even between wards in a single hospital. For hospitals with a relatively short length of stay, the screening strategies investigated in this study will result in lower cost savings and lower net benefits than shown in figure 6.

For a more comprehensive determination of cost-effectiveness from the societal perspective, more data is needed on the value of averted infections in terms of the additional survival, quality of life and the costs of MRSA infection, during hospital stay as well as after discharge. One would also hope to include the potential negative effects of isolation on quality of care [33] and possibly the costs of damage to hospital reputations or subsequent litigations. Yet, with the aforementioned limitations, this analysis provides a robust estimate of the costs of averting MRSA infection through screening and isolation. Our estimates can be considered in combination with the hospital’s own estimates, e.g. the additional costs of single room isolation and savings of averted infections, to support decision making on cost-effective infection control strategies.

### Conclusions

Based upon our simulation model, three important conclusions can be drawn related to MRSA admission screening:

(1) Excluding any financial benefits from averted infections, the choice of strategy depends on the setting, the costs of isolation and the hospital’s willingness to pay to avert infection. In both settings, selective screening with a chromogenic media-based test is the least costly strategy in terms of the cost per infection averted. More infections can be averted by replacing the chromogenic media-based test with a PCR test, at additional costs. The additional infections that can be averted with universal screening with PCR are relatively costly.

(2) The ranking of strategies is sensitive to additional daily costs of single room isolation. At thresholds of $45 and $106, in high and medium prevalence settings respectively, selective screening with PCR becomes dominant over selective chromogenic media-based screening.

(3) Assuming $17,645 benefit per infection averted, all evaluated strategies using base-case assumptions are cost-saving with the exception of universal screening with PCR in a medium prevalence setting. The most cost-saving strategies in high and medium prevalence settings are selective screening with PCR and selective screening with a chromogenic media based test, respectively.

## References

[1] Tiemersma EW, Bronzwaer SL, Lyytikainen O, Degener JE, Schrijnemakers P (2004). Methicillin-resistant Staphylococcus aureus in Europe,
1999-2002.. Emerg Infect Dis.

[2] Panlilio AL, Culver DH, Gaynes RP, Banerjee S, Henderson TS (1992). Methicillin-resistant Staphylococcus aureus in U.S. hospitals,
1975-1991.. Infect Control Hosp Epidemiol.

[3] Klein E, Smith DL, Laxminarayan R (2007). Hospitalizations and deaths caused by methicillin-resistant
Staphylococcus aureus, United States, 1999-2005.. Emerg Infect Dis.

[4] Klevens RM, Morrison MA, Nadle J, Petit S, Gershman K (2007). Invasive methicillin-resistant Staphylococcus aureus infections
in the United States.. JAMA.

[5] Harbarth S, Rutschmann O, Sudre P, Pittet D (1998). Impact of methicillin resistance on the outcome of patients with
bacteremia caused by Staphylococcus aureus.. Arch Intern Med.

[6] Cosgrove SE, Qi Y, Kaye KS, Harbarth S, Karchmer AW (2005). The impact of methicillin resistance in Staphylococcus aureus
bacteremia on patient outcomes: mortality, length of stay, and hospital
charges.. Infect Control Hosp Epidemiol.

[7] Health Council of the Netherlands (2006). MRSA policy in the Netherlands.. The Hague: Health Council of the Netherlands, 2006 publication no
2006/17.

[8] Bootsma MC, Diekmann O, Bonten MJ (2006). Controlling methicillin-resistant Staphylococcus aureus:
quantifying the effects of interventions and rapid diagnostic
testing.. Proc Natl Acad Sci U S A.

[9] Chaix C, Durand-Zaleski I, Alberti C, Brun-Buisson C (1999). Control of endemic methicillin-resistant Staphylococcus aureus: a
cost-benefit analysis in an intensive care unit.. JAMA.

[10] Harbarth S, Fankhauser C, Schrenzel J, Christenson J, Gervaz P (2008). Universal screening for methicillin-resistant Staphylococcus
aureus at hospital admission and nosocomial infection in surgical
patients.. JAMA.

[11] Herr CE, Heckrodt TH, Hofmann FA, Schnettler R, Eikmann TF (2003). Additional costs for preventing the spread of
methicillin-resistant Staphylococcus aureus and a strategy for reducing
these costs on a surgical ward.. Infect Control Hosp Epidemiol.

[12] Keshtgar MR, Khalili A, Coen PG, Carder C, Macrae B (2008). Impact of rapid molecular screening for meticillin-resistant
Staphylococcus aureus in surgical wards.. Br J Surg.

[13] Ritchie K, Bradbury I, Craig J, Eastgate J, Foster L (2007). The clinical and cost effectiveness of screening for
meticillin-resistant *Staphylococcus aureus*
(MRSA).

[14] Robicsek A, Beaumont JL, Paule SM, Hacek DM, Thomson RB (2008). Universal surveillance for methicillin-resistant Staphylococcus
aureus in 3 affiliated hospitals.. Ann Intern Med.

[15] Wernitz MH, Keck S, Swidsinski S, Schulz S, Veit SK (2005). Cost analysis of a hospital-wide selective screening programme
for methicillin-resistant Staphylococcus aureus (MRSA) carriers in the
context of diagnosis related groups (DRG) payment.. Clin Microbiol Infect.

[16] Gould IM (2006). Costs of hospital-acquired methicillin-resistant Staphylococcus
aureus (MRSA) and its control.. Int J Antimicrob Agents.

[17] Gold MR, Siegel JE, Russell LB, Weinstein MC (1996). Cost-effectiveness in Health and Medicine..

[18] Albrich WC, Harbarth S (2008). Health-care workers: source, vector, or victim of
MRSA?. Lancet Infect Dis.

[19] Coello R, Glynn JR, Gaspar C, Picazo JJ, Fereres J (1997). Risk factors for developing clinical infection with
methicillin-resistant Staphylococcus aureus (MRSA) amongst hospital patients
initially only colonized with MRSA.. J Hosp Infect.

[20] Cepeda JA, Whitehouse T, Cooper B, Hails J, Jones K (2005). Isolation of patients in single rooms or cohorts to reduce spread
of MRSA in intensive-care units: prospective two-centre
study.. Lancet.

[21] Grundmann H, Hori S, Winter B, Tami A, Austin DJ (2002). Risk factors for the transmission of methicillin-resistant
Staphylococcus aureus in an adult intensive care unit: fitting a model to
the data.. J Infect Dis.

[22] Hori S, Sunley R, Tami A, Grundmann H (2002). The Nottingham Staphylococcus aureus population study: prevalence
of MRSA among the elderly in a university hospital.. J Hosp Infect.

[23] Nijssen S, Bonten MJ, Weinstein RA (2005). Are active microbiological surveillance and subsequent isolation
needed to prevent the spread of methicillin-resistant Staphylococcus
aureus?. Clin Infect Dis.

[24] Jarvis WR, Schlosser J, Chinn RY, Tweeten S, Jackson M (2007). National prevalence of methicillin-resistant Staphylococcus
aureus in inpatients at US health care facilities, 2006.. Am J Infect Control.

[25] Rubin RJ, Harrington CA, Poon A, Dietrich K, Greene JA (1999). The economic impact of Staphylococcus aureus infection in New
York City hospitals.. Emerg Infect Dis.

[26] Engemann JJ, Carmeli Y, Cosgrove SE, Fowler VG, Bronstein MZ (2003). Adverse clinical and economic outcomes attributable to
methicillin resistance among patients with Staphylococcus aureus surgical
site infection.. Clin Infect Dis.

[27] Smith DL, Levin SA, Laxminarayan R (2005). Strategic interactions in multi-institutional epidemics of
antibiotic resistance.. Proc Natl Acad Sci U S A.

[28] Luteijn JM, Hubben GA, Pechlivanoglou P, Bonten MJ, Postma MJ (2010). Diagnostic accuracy of culture-based and PCR-based detection
tests for MRSA - a meta-analysis.. Clin Microbiol Infect.

[29] Wigglesworth N, Wilcox MH (2006). Prospective evaluation of hospital isolation room
capacity.. J Hosp Infect.

[30] Chaudhury H, Mahmood A, Valente M (2005). Advantages and disadvantages of single-versus multiple-occupancy
rooms in acute care environments: A review and analysis of the
literature.. Environment and Behavior.

[31] Conterno LO, Shymanski J, Ramotar K, Toye B, Zvonar R (2007). Impact and cost of infection control measures to reduce
nosocomial transmission of extended-spectrum beta-lactamase-producing
organisms in a non-outbreak setting.. J Hosp Infect.

[32] Clements A, Halton K, Graves N, Pettitt A, Morton A (2008). Overcrowding and understaffing in modern health-care systems: key
determinants in meticillin-resistant Staphylococcus aureus
transmission.. Lancet Infect Dis.

[33] Stelfox HT, Bates DW, Redelmeier DA (2003). Safety of patients isolated for infection
control.. JAMA.

[34] Paule SM, Hacek DM, Kufner B, Truchon K, Thomson RB (2007). Performance of the BD GeneOhm methicillin-resistant
Staphylococcus aureus test before and during high-volume clinical
use.. J Clin Microbiol.

[35] Lagace-Wiens PR, Alfa MJ, Manickam K, Harding GK (2008). Reductions in workload and reporting time by use of
methicillin-resistant Staphylococcus aureus screening with MRSASelect medium
compared to mannitol-salt medium supplemented with
oxacillin.. J Clin Microbiol.

[36] Woolhandler S, Campbell T, Himmelstein DU (2003). Costs of health care administration in the United States and
Canada.. N Engl J Med.

